# CRISPR Interference Reveals That All-*Trans*-Retinoic Acid Promotes Macrophage Control of Mycobacterium tuberculosis by Limiting Bacterial Access to Cholesterol and Propionyl Coenzyme A

**DOI:** 10.1128/mbio.03683-21

**Published:** 2022-01-18

**Authors:** Gregory H. Babunovic, Michael A. DeJesus, Barbara Bosch, Michael R. Chase, Thibault Barbier, Amy K. Dickey, Bryan D. Bryson, Jeremy M. Rock, Sarah M. Fortune

**Affiliations:** a Department of Immunology and Infectious Diseases, Harvard T. H. Chan School of Public Health, Boston, Massachusetts, USA; b Laboratory of Host-Pathogen Biology, The Rockefeller University, New York, New York, USA; c Division of Pulmonary and Critical Care Medicine, Massachusetts General Hospital, Boston, Massachusetts, USA; d Department of Biological Engineering, Massachusetts Institute of Technology, Cambridge, Massachusetts, USA; e Ragon Institute of MGH, MIT, and Harvard, Cambridge, Massachusetts, USA; National Institute of Allergy and Infectious Diseases

**Keywords:** CRISPR interference, *Mycobacterium tuberculosis*, cholesterol, macrophages, nutritional immunity, propionyl-CoA, retinoic acid

## Abstract

Macrophages are a protective replicative niche for Mycobacterium tuberculosis (Mtb) but can kill the infecting bacterium when appropriately activated. To identify mechanisms of clearance, we compared levels of bacterial restriction by human macrophages after treatment with 26 compounds, including some currently in clinical trials for tuberculosis. All-*trans*-retinoic acid (ATRA), an active metabolite of vitamin A, drove the greatest increase in Mtb control. Bacterial clearance was transcriptionally and functionally associated with changes in macrophage cholesterol trafficking and lipid metabolism. To determine how these macrophage changes affected bacterial control, we performed the first Mtb CRISPR interference screen in an infection model, identifying Mtb genes specifically required to survive in ATRA-activated macrophages. These data showed that ATRA treatment starves Mtb of cholesterol and the downstream metabolite propionyl coenzyme A (propionyl-CoA). Supplementation with sources of propionyl-CoA, including cholesterol, abrogated the restrictive effect of ATRA. This work demonstrates that targeting the coupled metabolism of Mtb and the macrophage improves control of infection and that it is possible to genetically map the mode of bacterial death using CRISPR interference.

## INTRODUCTION

Although macrophages are specialized for the clearance of pathogens, they serve as the primary replicative niche for Mycobacterium tuberculosis (Mtb). One of the paradoxes in the field is that human macrophages display limited capacity to kill Mtb *in vitro* (1, 2), but during *in vivo* infection in nonhuman primates and by extension presumably in humans there is significant bacterial clearance (3). Many groups have proposed the existence of stimuli that activate at least some subsets of macrophages to kill Mtb *in vivo* and that these routes may be therapeutically leveraged to increase bacterial clearance; indeed, several activators can act on macrophages or their monocytic precursors to increase the control of Mtb infection (1, 2, 4–15). Some of these, including vitamin D and imatinib, have been moved into clinical trials as potential host-directed therapies (16–18). However, results from the largest vitamin D supplementation trials were disappointing (19, 20), and the piecemeal nature of macrophage activator discovery has obscured which activators are most potent in inducing Mtb control and under what conditions.

Efforts to develop macrophage-focused host-directed therapies for Mtb have been hampered because we lack a fundamental understanding of how macrophages kill Mtb. A range of macrophage effector mechanisms have been associated with Mtb control, including oxidative attack, cell wall perturbation, and starvation (1, 6, 12, 15, 21). However, the bacterium is remarkably resistant to all of these stressors *in vitro*, and it remains unclear which macrophage effectors cause bacterial death during infection. This knowledge gap is aggravated by the paucity of tools to study essential bacterial processes, which are presumably the foundation of bacterial life and death *in vivo*.

In this study, we sought to identify the most effective way of activating human macrophages to kill Mtb and then systematically define the mechanism of bacterial death. Through a head-to-head comparison of a panel of macrophage activators in primary human macrophages from multiple donors, we found that treatment with all-*trans*-retinoic acid (ATRA) was the most effective means of activating human macrophages to restrict Mtb infection. We combined host- and pathogen-centric genomic tools, including meso-scale bacterial CRISPR inhibition screening, to define the mechanism of bacterial clearance under this activating condition. This systems-level analysis demonstrated that ATRA treatment limits bacterial access to cholesterol, resulting in propionyl coenzyme A (propionyl-CoA) limitation and stress on the anaplerotic and gluconeogenic pathways fed by this metabolite in Mtb.

## RESULTS

### Comparing macrophage activator activities against Mtb.

We sought to identify the most robust activators of human macrophage activity against Mtb, defined as those most capable of driving bacterial clearance in both classically and alternatively activated macrophages from multiple human donors. We performed a head-to-head assessment of 26 cytokines and small molecules that have previously been described as impacting monocyte/macrophage inflammatory phenotype or control of Mtb. We quantitatively compared their abilities to drive Mtb control in human primary monocyte-derived macrophages derived through both macrophage colony-stimulating factor (MO-MCSF) and granulocyte-MCSF (MO-GMCSF) maturation. To quantify bacterial clearance, we used an autobioluminescent strain of Mtb which enabled us to measure changes in bacterial load over time (22, 23). Luminescence in this strain is dependent on bacterial number and metabolic state, providing a sensitive measure of bacterial control and a precise tool for comparative analysis.

**FIG 1:**
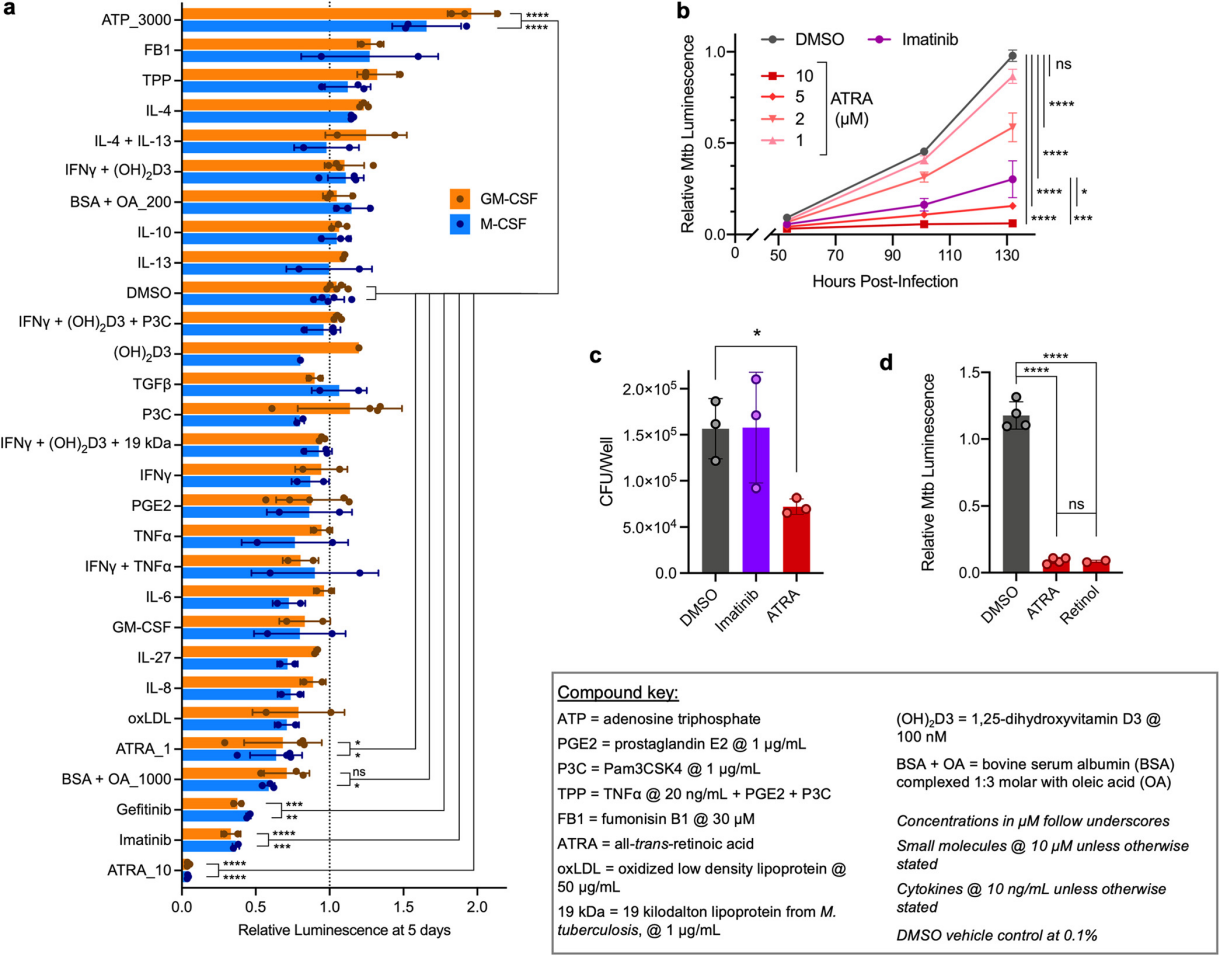
All-*trans*-retinoic acid outperforms other activators in eliciting human macrophage control of M. tuberculosis infection. (a) Control by differentially matured MO-MCSF and MO-GMCSF (each point represents 1 donor) of autobioluminescent Mtb H37Rv at 5 days following infection and treatment with different activators, detailed in the compound key. (b) Growth in MO-GMCSF (3 donors) of autobioluminescent Mtb over time following treatment with different concentrations of ATRA or imatinib at 10 μM. (c) Comparison of Mtb loads in MO-GMCSF (3 donors) by CFU assay for ATRA and imatinib (10 μM) at 5 days. (d) Growth of autobioluminescent Mtb in MO-GMCSF (2 to 4 donors) at 5 days following treatment with ATRA or retinol (10 μM). All infections were performed at a multiplicity of infection of 2 bacteria per macrophage, with between 2 and 5 donors (a, c, d) or 3 donors (b) per condition. All summary data represent means ± standard deviations (SD). Any comparisons to the DMSO control not marked with statistical significance were not significant; statistics were performed using a 2-way analysis of variance (ANOVA) with Dunnett’s multiple-comparison test (a), an ordinary one-way ANOVA on area under the curve measurements with Šídák’s multiple-comparison test (b), a repeated-measures ANOVA with Dunnett’s multiple-comparison test (c), or an ordinary one-way ANOVA with Tukey’s multiple-comparison test (d). *, *P* < 0.05; **, *P* < 0.01; ***, *P* < 0.001; ****, *P* < 0.0001.

Two classes of molecules drove the greatest reduction in bacterial viability in both MO-MCSF and MO-GMCSF across multiple donors (Fig. 1a). The first class was the tyrosine kinase inhibitors imatinib and gefitinib, which have shown such promise that imatinib is currently in clinical trials as a host-directed therapy (7, 17). The second was all-*trans*-retinoic acid (ATRA), which outperformed equimolar imatinib in driving dose-dependent bacterial control as measured in both the luminescence assay (Fig. 1b) and confirmatory CFU assays (Fig. 1c). Bacterial restriction was macrophage dependent, as ATRA has no impact on Mtb growth in axenic culture (see Fig. S1 in the supplemental material). ATRA is an active metabolite of vitamin A (retinol), which also increased bacterial control (Fig. 1d).

10.1128/mbio.03683-21.2 FIG S1 ATRA does not inhibit Mtb growth in axenic broth culture. Autobioluminescent Mtb H37Rv was grown in complete 7H9 and treated with 10 μM ATRA (3 separate cultures). Growth was measured over time using optical density (a) and bacterial luminescence (b). Data represent means ± SD. Download FIG S1, PDF file, 0.1 MB. Copyright © 2022 Babunovic et al. 2022 Babunovic et al. https://creativecommons.org/licenses/by/4.0/ This content is distributed under the terms of the Creative Commons Attribution 4.0 International license.

### Associating macrophage processes with ATRA-mediated control.

**FIG 2:**
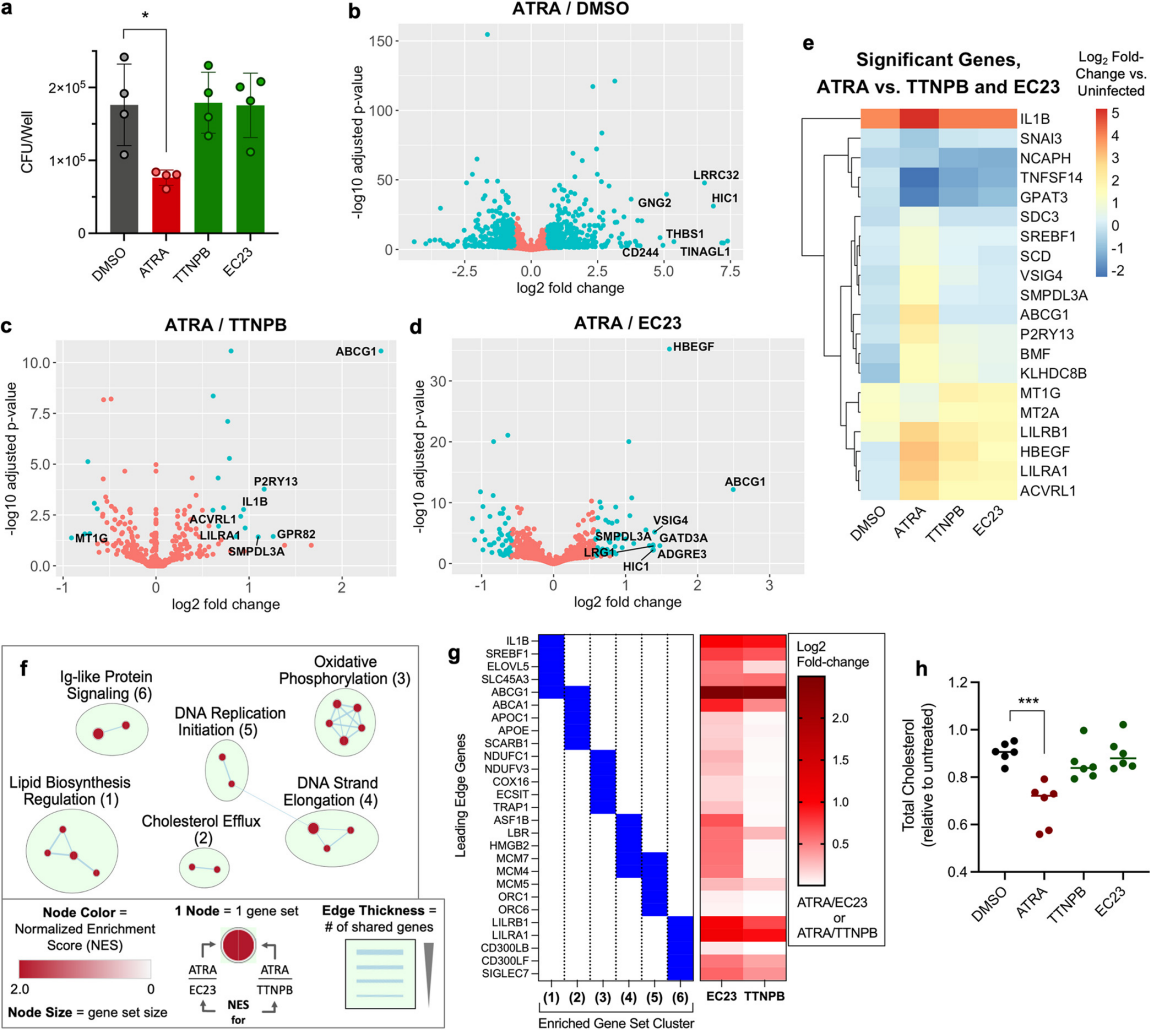
Macrophage restriction of M. tuberculosis via all-*trans*-retinoic acid is associated with cholesterol limitation. (a) Comparison of Mtb loads in MO-GMCSF (3 donors) by CFU assay for ATRA and other pan-RAR agonists. (b to d) Volcano plots of differential gene expression by Mtb-infected MO-GMCSF (3 donors), comparing ATRA to a DMSO control (b) or to nonrestrictive pan-RAR agonists (c, d). (e) Clustered heatmap of gene expression relative to that in uninfected macrophages for all genes significantly differentially expressed during ATRA treatment relative to treatment with both nonrestrictive RAR agonists in infected MO-GMCSF. (f) Network plot of gene set clusters, the expression of which was significantly enriched during ATRA treatment relative to treatment with both of the nonrestrictive RAR agonists, in Mtb-infected MO-GMCSF. (g) Top 5 genes (largest absolute fold change) driving the enrichment of each gene set cluster shown in panel f and their differential expression values from the data displayed in panels c and d. (h) Changes in total cellular cholesterol 1 day after Mtb infection and treatment for MO-GMCSF (6 donors) treated with ATRA or nonrestrictive RAR agonists. All infections were performed at a multiplicity of infection of 2 bacteria per macrophage. All compounds were used at 10 μM in 0.2% DMSO. (a, h) Any comparisons to the DMSO control not marked with statistical significance were not significant in summary data representing means ± SD (a) and in lines representing the data median (h). Statistics were performed using a repeated-measures one-way ANOVA with Dunnett’s multiple-comparison test (a) or an ordinary one-way ANOVA with Dunnett’s multiple-comparison test (h). *, *P* < 0.05; **, *P* < 0.01; ***, *P* < 0.001; ****, *P* < 0.0001.

ATRA is used to treat acute promyelocytic leukemia (24), and its clinical relevance has stimulated the development of other retinoic acid receptor (RAR) agonists. EC23 and TTNPB are two agonists with pan-RAR activity similar to that of ATRA (25, 26), which we initially tested in our assays because they are light stable. As expected, we found that ATRA, EC23, and TTNPB induced the expression of RAR-responsive genes to a similar extent (Fig. S2). However, EC23 and TTNPB were less able to drive MO-GMCSF restriction of Mtb than ATRA at equivalent concentrations (Fig. 2a).

10.1128/mbio.03683-21.3 FIG S2 ATRA and other pan-RAR agonists equivalently activate downstream genes. THP1 monocytes were differentiated into macrophage-like cells by treatment with PMA and then treated overnight with the indicated compounds at 10 μM prior to RNA extraction and RT-qPCR for the indicated targets (3 measurements). Expression was calculated relative to that of GAPDH and then further compared to the DMSO vehicle condition. Lines indicate median values; statistics were performed using ordinary one-way ANOVA with Dunnett’s multiple-comparison test. *, *P* < 0.05. Download FIG S2, PDF file, 0.1 MB. Copyright © 2022 Babunovic et al. 2022 Babunovic et al. https://creativecommons.org/licenses/by/4.0/ This content is distributed under the terms of the Creative Commons Attribution 4.0 International license.

We leveraged the differences between these very similar RAR agonists to identify changes in macrophage biology associated specifically with the ATRA-mediated restriction of Mtb. We performed transcriptome sequencing (RNA-seq) on infected MO-GMCSF treated with ATRA, TTNPB, or EC23 as well as sham-treated and uninfected cells. ATRA treatment led to the differential expression of thousands of genes (Fig. 2b and Table S1), but relatively few genes between ATRA and TTNPB, ATRA and EC23, or ATRA and both nonrestrictive RAR agonists (defined as a >1.5 absolute fold change in mean expression; adjusted *P*, <0.05) (Fig. 2c to e and Table S1). We performed gene set enrichment and network analyses for the ATRA/TTNPB and ATRA/EC23 comparisons to define gene sets enriched specifically under the ATRA-treated condition relative to treatment with both of the nonrestrictive RAR agonists. We identified several clusters of gene sets, including oxidative phosphorylation, cholesterol efflux, and lipid biosynthesis regulation, as well as markers of macrophage replication and Ig-like protein signaling (Fig. 2f).

10.1128/mbio.03683-21.7 TABLE S1 RNA-seq data. Raw count data and DESeq2 analysis results associated with Fig. 2 are displayed in separate sheets. Download Table S1, XLSX file, 7.4 MB. Copyright © 2022 Babunovic et al. 2022 Babunovic et al. https://creativecommons.org/licenses/by/4.0/ This content is distributed under the terms of the Creative Commons Attribution 4.0 International license.

To define the processes that functionally contribute to macrophage control, we measured phenotypic correlates of the differential transcriptional responses, focusing first on those previously associated with bacterial restriction. Changes in oxidative phosphorylation are linked with aerobic glycolysis and reactive nitrooxidative species production, which follows Mtb infection of macrophages (27). Seahorse extracellular flux analysis of MO-GMCSF infected with Mycobacterium bovis BCG, a close relative of Mtb, showed that ATRA reduces the maximal respiratory capacity; however, the nonrestrictive RAR agonist TTNPB had indistinguishable effects (Fig. S3).

10.1128/mbio.03683-21.4 FIG S3 Both ATRA and TTNPB reduce macrophage maximal respiratory capacity. MO-GMCSF from 3 donors were infected with M. bovis BCG at a multiplicity of infection of 2 bacteria per macrophage and treated for 2 days with ATRA or TTNPB at 10 μM, with a baseline of 0.2% DMSO. Cells were prepared and analyzed with a Seahorse Mito stress test kit as per the manufacturer’s instructions. Data are shown for individual time points (a) or averaged across all 3 time points for each measurement (b). Data in panel a represent means ± SD. Statistics were performed using an ordinary two-way ANOVA with Tukey’s multiple-comparison test. ****, *P* < 0.0001; ns, *P* > 0.05. Download FIG S3, PDF file, 0.2 MB. Copyright © 2022 Babunovic et al. 2022 Babunovic et al. https://creativecommons.org/licenses/by/4.0/ This content is distributed under the terms of the Creative Commons Attribution 4.0 International license.

We next assessed cholesterol efflux and lipid biosynthesis regulation, which have also been associated with Mtb control by macrophages (5, 28, 29). These enrichments were driven by the largest gene expression changes between ATRA and the nonrestrictive RAR agonists, especially for the cholesterol efflux transporter ABCG1 (Fig. 2g). To test the specific effects of ATRA on cholesterol efflux and lipid biosynthesis regulation, we measured total cholesterol early during Mtb infection of MO-GMCSF. We observed a loss of cellular cholesterol upon ATRA treatment that did not occur upon treatment with TTNPB or EC23 (Fig. 2h), functionally associating cholesterol limitation with ATRA-mediated macrophage Mtb restriction.

Previous work identified ATRA-induced cholesterol reduction in infected monocytes and found that anti-HIV protease inhibitors, which dampened this cholesterol phenotype, also reversed ATRA-mediated Mtb restriction (5). Mechanistically, this study proposed that ATRA increases lysosomal acidification via NPC2-mediated reduction in lysosomal cholesterol, thereby promoting Mtb control. Other research has tied ATRA efficacy to autophagy (6), and targeting cholesterol biosynthesis with statins reduces Mtb growth through an autophagy-dependent mechanism (30, 31). These processes may be related, as cholesterol accumulation in macrophages during Mtb infection can interfere with phagolysosomal maturation and autophagic clearance of mycobacteria (32, 33). However, cholesterol is also a primary carbon source for Mtb and serves as a precursor for the synthesis of polyketide lipids, which are important for mycobacterial virulence (34, 35). Mtb requires cholesterol import and catabolism to survive in macrophages (35–37). Therefore, while the importance of cholesterol limitation in the antitubercular effect of ATRA is clear, it is unknown through what pathways this lipid restriction leads to bacterial control.

### Using bacterial CRISPRi to identify mechanisms of macrophage control.

Given the diverse possible mechanisms by which altered cholesterol and lipid metabolism might impact Mtb survival, we sought to genetically map the mechanism of bacterial restriction in the setting of ATRA treatment. We reasoned that during macrophage activation by sublethal doses of ATRA, there would be an increased requirement for Mtb genes that are specifically required for the organism to adapt to the more stringent host environment. This use of comparative gene essentiality to reveal *in vivo* bacterial vulnerabilities underpins previous transposon insertion sequencing (Tn-seq) screens, which have defined virulence pathways required under specific host conditions (38, 39). However, Tn-seq is limited in its ability to probe pathways that are essential *in vitro*, including many metabolic pathways, because these genes are not represented in transposon libraries. As we postulated that ATRA might impose core metabolic stresses on the bacterium, we instead leveraged our ability to inducibly silence gene expression in Mtb using CRISPR interference (CRISPRi) (40). CRISPRi uses a catalytically inactive form of Cas9 (dCas9), which when paired with a single guide RNA (sgRNA) can be targeted to any site in the Mtb genome containing a simple protospacer-adjacent motif (PAM) sequence; dCas9 inhibits transcription of the targeted gene or operon. CRISPRi allows tunable inhibition of essential bacterial processes, and customized sgRNA libraries permit targeted testing of pathways most relevant to infection.

We constructed an sgRNA library targeting 110 genes implicated in central carbon metabolism, essential biosynthetic processes, known virulence pathways, and stress response systems (Table 1 and Table S2). By targeting only specific pathway members, we constrained the size of the library, enabling pathway mapping in primary cells across multiple conditions. When targeting genes that are essential for *in vitro* growth (41), we used sgRNAs selected to silence gene function to different degrees (42), thereby generating a diversity of genetic hypomorphs in our final library. Graded silencing (with strong, medium, and weak sgRNAs for each essential gene, depending on its vulnerability to knockdown) is important for identifying quantitative changes in selection. We also included an overrepresented set of negative sgRNAs that do not target any site in the Mtb genome as controls for downstream analysis.

**TABLE 1:** Genes included in the M. tuberculosis CRISPRi library

Gene (reference[s])^*a*^	Function^*b*^
***metE***	Metabolite biosynthesis and import
*metH*	
***panC***	
***trpB***	
*ansA*	
***ilvB1***	
***glnA1***	
***gltB***	
***asnB***	
***nadB***	
***ilvE***	
***serB2***	
*sugC*	
*lipB*	
***csd***	
***iscS***	
***ino1***	
***pabC***	
***pheA***	
***purF***	
***ilvD***	
***leuD***	
***hemE***	
***metA***	
*phoT*	
*pstS3*	
*bioB*	
*guaB1*	
*phoP*	Virulence and stress response
*Rv0818 (*82)	
*hrcA* (83)	
*eccB1*	
*ureC* (84)	
*mycP1* (45)	
***pptT***	Virulence lipid synthesis and transport
*fadD26*	
*ppsD*	
*ppsE*	
*drrA*	
*drrB*	
*mas*	
*katG*	Oxidative- and nitrosative-stress response
*lpdC (85)	
*hoaS (44)	
*ahpC* (44, 85)	
*dlaT (44)	
*bpoC*	
*aceE (44)	
*sseA* (39)	
***ribA2***	Flavin metabolism
***aroF***	
***ribF***	
*mmaA4*	Cell wall biogenesis and function
*Rv0805* (86)	
*mmpl11*	
*cysQ*	Sulfurylation
*birA*	Biotinylation
***pgi***	Glycolysis/gluconeogenesis
*fba*	
***tpi***	
***pykA***	
*ppgK*	
*pfkA*	
***pgk***	
***sucC***	TCA cycle
***acn***	
*gdh*	
*icd1*	
*icd2*	
*mdh*	
***gnd2***	Pentose phosphate pathway
***tkt***	
*zwf1*	
*icl1*	Glyoxylate shunt
***glcB***	
*aceAa*	
*ramB* (87)	
*prpD*	Methylcitrate cycle
*prpR*	
***accA3***	Methylmalonyl pathway
*mutA*	
*ppdK*	Anaplerotic reactions
*pckA*	
*mez*	
*lldD2*	Pyruvate and lactate metabolism
* ***lpdC***	
*aceE	
*hoaS (44)	Glutamate catabolism
*dlaT (44)	
*glpK*	Glycerol metabolism
*glpD1*	
*mce1C*	Lipid import and catabolism
*cpsA* (47)	
*fadD31*	
*mce4A*	
*mce4B*	
*hsaD*	
***menH***	Oxidative phosphorylation
***atpA***	
*cydD*	
*mctB*	Metal ion transport and response
*mbtA*	
*mbtB*	
*zur*	
*furA*	
*smtB* (88)	
*ideR*	
*irtA*	
*irtB*	
*mmpL4*	
***hupB***	Nucleosome structure
*glgP*	Glycogen catabolism
*Rv1421*	Unknown
*Rv1780*	

a Genes in bold are essential by Tn-seq analysis for Mtb growth in standard media (41). Genes with asterisks are present in multiple categories.

b The functions from “Glycolysis/gluconeogenesis” to “Lipid import and catabolism” have to do with carbon metabolism.

**FIG 3:**
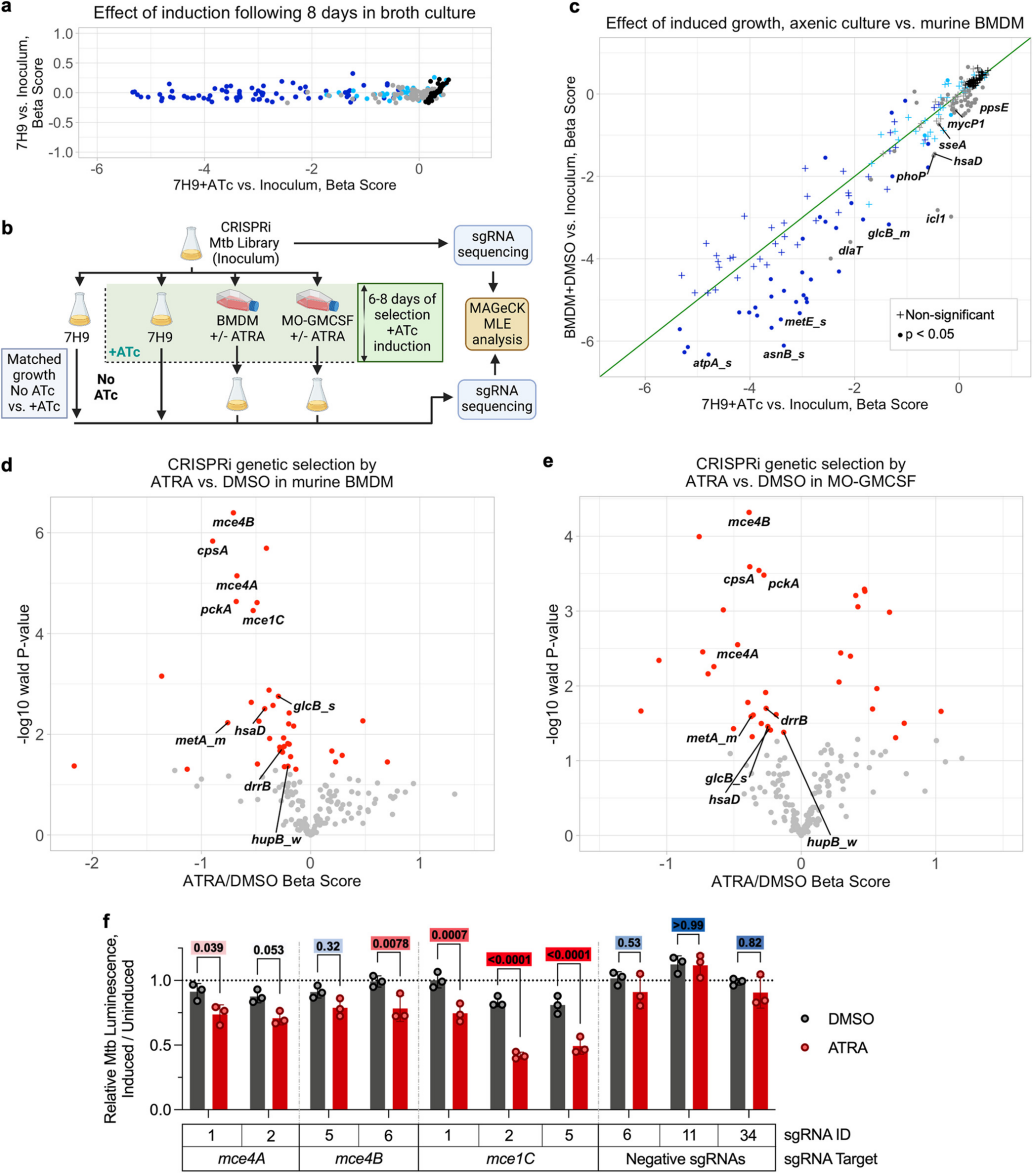
Bacterial CRISPRi reveals increased reliance of M. tuberculosis on lipid import genes in macrophages treated with all-*trans*-retinoic acid. (a) Changes in gene-sgRNA set representation as measured by beta score (similar to log fold change), following CRISPRi library induction (+ATc) in axenic medium compared to the score for uninduced growth. Dark-blue points represent essential genes targeted with sets of strong (_s) or medium (_m) hypomorph sgRNAs (3 sgRNAs per point), light-blue points represent essential genes targeted with weak (_w) hypomorph sets of sgRNAs (3 sgRNAs per point), gray points represent nonessential genes, and black points represent individual negative (nontargeting) sgRNAs. (b) Schematic of CRISPRi experiments in macrophages and axenic broth culture. (c) Changes in gene-sgRNA set representation as measured by the beta score following CRISPRi library induction in axenic medium compared to the score for induced (+ATc) growth in primary mouse macrophages. Significance is measured by determining the Wald *P* value (equivalent to an adjusted *P* value). Point colors are as described for panel a; the green line is at the diagonal. (d, e) Volcano plots showing changes in gene-sgRNA set representation between 5 μM ATRA- and DMSO-treated primary macrophages from mouse (d) and human (e) (MO-GMCSF) sources. Red points represent gene-sgRNA sets that are significant at a Wald *P* value cutoff of <0.05. (f) MO-GMCSF control of autobioluminescent single-sgRNA clonal strains of Mtb at 5.5 days following infection and treatment with 5 μM ATRA, comparing strains with ATc-induced knockdown of the indicated Mtb genes to the same strains with no knockdown. Statistics were performed using a 2-way ANOVA with Šídák’s multiple-comparison test; color-coded adjusted *P* values are shown above each comparison (red indicates a *P* of <0.05, white indicates a *P* of 0.05, and blue indicates a *P* of >0.05 [continuous scale]). Infections were performed at a multiplicity of infection of 1 bacterium (a to e) or 2 bacteria (f) per macrophage.

10.1128/mbio.03683-21.8 TABLE S2 sgRNA list. All sgRNAs targeting each gene in the CRISPRi library are listed, along with (for essential genes) the strength of knockdown as determined from sgRNA dropout in induced axenic culture. Associated results are shown in Fig. 3 and 5. Download Table S2, XLSX file, 0.04 MB. Copyright © 2022 Babunovic et al. 2022 Babunovic et al. https://creativecommons.org/licenses/by/4.0/ This content is distributed under the terms of the Creative Commons Attribution 4.0 International license.

### CRISPRi identifies requirements for Mtb intramacrophage growth.

CRISPRi knockdowns performed as anticipated in axenic culture. Strong and medium hypomorphs of essential genes became underrepresented in the population to the greatest extent, followed by weak hypomorphs of essential genes, sgRNAs targeting nonessential genes, and finally negative sgRNAs, which showed no depletion upon CRISPRi induction (Fig. 3a). Importantly, we did not see a loss of attenuating guides in uninduced cultures, indicating that the expression of CRISPRi components is tightly controlled, as we have previously described (40).

To date, the Mtb CRISPRi screening system has not been assessed in any model of infection. Therefore, we optimized infection methodology in murine bone marrow-derived macrophages (BMDM) (Fig. 3b); ATRA drives Mtb restriction in BMDM to an extent similar to that in human macrophages (Fig. S4). We compared the relative levels of depletion of library members upon induction between BMDM and axenic culture to confirm that genes previously described as essential for the intramacrophage growth of Mtb are indeed more required in this system (Fig. 3c and Table S3). As expected, given prior Tn-seq screens and directed analyses, genes involved in resistance to acid (*phoP*) (43) and nitrooxidative stress (*dlaT*) (44) are more required for growth in macrophages than *in vitro*, as are metabolic enzymes necessary for growth on cholesterol (*icl1*, *hsaD*) (34) and genes required for early secretory antigenic target (6 kDa) (ESX) secretion (*eccB1*, *mycP1*) (45).

10.1128/mbio.03683-21.5 FIG S4 ATRA is effective at limiting Mtb growth in BMDM. BMDM (*n* = 3) were infected with autobioluminescent Mtb at a multiplicity of infection of 2 bacteria per macrophage and treated with ATRA at various concentrations or imatinib at 10 μM, with a baseline of 0.1% DMSO. Statistics were performed using an ordinary one-way ANOVA on area under the curve measurements with Šídák’s multiple-comparison test. *, *P* < 0.05; **, *P* < 0.01; ***, *P* < 0.001; ****, *P* < 0.0001. Download FIG S4, PDF file, 0.1 MB. Copyright © 2022 Babunovic et al. 2022 Babunovic et al. https://creativecommons.org/licenses/by/4.0/ This content is distributed under the terms of the Creative Commons Attribution 4.0 International license.

10.1128/mbio.03683-21.9 TABLE S3 CRISPRi data. Raw count data and MAGeCK analysis results associated with Fig. 3 and 5 are displayed in separate sheets. Individual sgRNAs with no reads across all conditions (as observed in the raw count sheet) were excluded from MAGeCK analysis. Figure 3a plots results from experiment 1/analysis 1; Fig. 3c plots results from experiment 1/analysis 1 and analysis 2, with significance values from analysis 3; Fig. 3d plots results from experiment 1/analysis 4; Fig. 3e plots results from experiment 2; Fig. 5 includes results from experiment 1/analysis 4 and experiment 2. Download Table S3, XLSX file, 0.2 MB. Copyright © 2022 Babunovic et al. 2022 Babunovic et al. https://creativecommons.org/licenses/by/4.0/ This content is distributed under the terms of the Creative Commons Attribution 4.0 International license.

The use of graded hypomorphic CRISPRi knockdowns targeting essential genes also revealed novel phenotypes, which were especially notable for a few core metabolic processes. For example, we observed a reduced requirement for the essential quinone synthase *menH* in macrophages compared to the requirement in axenic culture, indicating that Mtb within BMDM is less dependent on the electron transport chain for growth and survival. In addition, previous studies have found that the methionine synthase genes *metE* and *metH* are differentially required depending on vitamin B_12_ availability; *metE* is essential in standard culture media without vitamin B_12_ (41, 46). Our library targeted both genes, revealing increased *metE* essentiality in BMDM (Fig. 3c) but no effect of silencing *metH* (Table S3), suggesting that Mtb-accessible vitamin B_12_ is limited in BMDM.

### Bacteria are more reliant on lipid import and cholesterol catabolism in ATRA-treated than in untreated macrophages.

We then used bacterial CRISPRi to determine Mtb gene essentiality in macrophages with and without sublethal ATRA treatment. We identified 39 genes that were differentially required (*P* < 0.05) in BMDM and 38 genes that were differentially required in MO-GMCSF (Fig. 3d and e and Table S3) after ATRA treatment. These included genes implicated in central carbon metabolism, amino acid biosynthesis, and transmembrane transport. We did not identify canonical virulence genes as more required for survival in ATRA-treated macrophages. To better characterize specific host stresses that are key to ATRA efficacy across species, we focused on 9 Mtb genes that become significantly more essential in both MO-GMCSF and BMDM treated with ATRA (*mce4A*, *mce4B*, *cpsA*, *pckA*, *drrB*, *metA*, *glcB*, *hsaD*, and *hupB*) (Fig. 3d and e). Notably, this analysis did not identify genes involved in oxidative and nitrosative stress responses, such as *sseA* or *katG*, suggesting that this is not the primary stressor imposed on Mtb by ATRA treatment of macrophages.

These 9 key genes include those involved in cholesterol import (*mce4A*, *mce4B*) (35, 47) and catabolism (*hsaD*) (34). This suggests that ATRA treatment increases Mtb’s metabolic vulnerability to cholesterol limitation and is highly consistent with the transcriptional and functional alterations in cholesterol metabolism identified by macrophage analyses. We also observed an increased requirement for *cpsA*, a gene important for fatty acid (FA) uptake (47), and a similar pattern for *mce1C*, a component of the FA import machinery (48). We sought to validate a subset of these changes in single-strain experiments, focusing on the bacterial requirements for *mce4A*, *mce4B*, and *mce1C* and measuring bacterial fate using the luminescence reporter, providing an orthologous endpoint measure of bacterial fitness compared to that of the sequencing-based approach used in the primary screen. Consistent with the screening data, single-strain infection knockdowns of these genes resulted in greater attenuation in ATRA-treated than in dimethyl sulfoxide (DMSO) vehicle-treated MO-GMCSF (Fig. 3f).

### ATRA acts via cholesterol and propionyl-CoA starvation.

**FIG 4:**
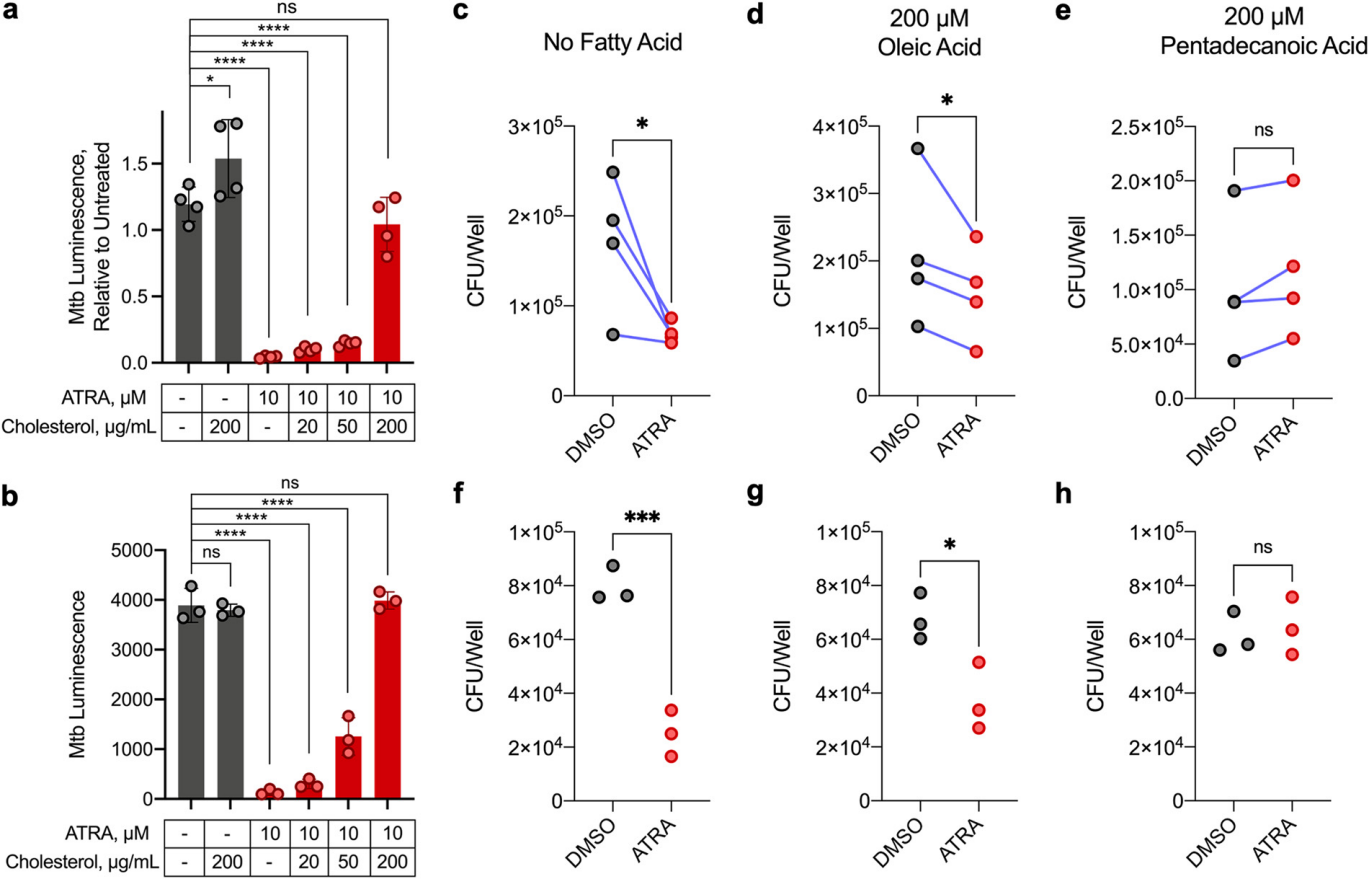
Macrophage restriction of M. tuberculosis elicited by all-*trans*-retinoic acid can be relieved by cholesterol or odd-chain fatty acids. (a, b) Control of autobioluminescent Mtb treated with ATRA and various concentrations of water-soluble cholesterol, in MO-GMCSF at 5.5 days (a) or in BMDM at 9.5 days (b). (c to h) Comparison of Mtb loads by CFU assay at 5 days in MO-GMCSF (c to e) or BMDM (f to h). ATRA was added at 10 μM, and fatty acids were added complexed to essentially fatty acid-free bovine serum albumin (BSA) at a 3:1 ratio. All infections were performed at a multiplicity of infection of 2 bacteria per macrophage, with all samples in triplicate (3 donors for MO-GMCSF). DMSO was used at 0.1% in all comparisons. Statistics were performed using ordinary one-way ANOVAs with Šídák’s (a) or Dunnett’s (b) multiple-comparison test, ratio paired *t* tests (c to e), or unpaired *t* tests (f to h). *, *P* < 0.05; **, *P* < 0.01; ***, *P* < 0.001; ****, *P* < 0.0001; ns, not significant.

These data demonstrate that ATRA treatment increases Mtb’s requirement for cholesterol import and catabolism. One plausible model, given the changes in macrophage cholesterol metabolism that we identified, is that in ATRA-treated macrophages Mtb becomes starved for these key carbon sources. Consistent with this, supplementation of Mtb-infected macrophages with soluble free cholesterol completely abrogated the antimicrobial effect of ATRA (Fig. 4a and b).

The increased requirement for *mce1C* and *cpsA* may indicate the increased essentiality of FA import, or it may be a result of linked cholesterol and FA transport in Mtb, mediated by shared functional and regulatory subunits of the Mce4 and Mce1 complexes (48). To determine the relevance of FA starvation, we tested the restrictive effect of ATRA in macrophages supplemented with different FAs (Fig. 4c to h). Supplementation with the even-chain FA (ECFA) oleic acid did not relieve ATRA-mediated control. However, ATRA’s effect was nullified by the odd-chain FA (OCFA) pentadecanoic acid used at the same molar concentration as oleic acid.

**FIG 5:**
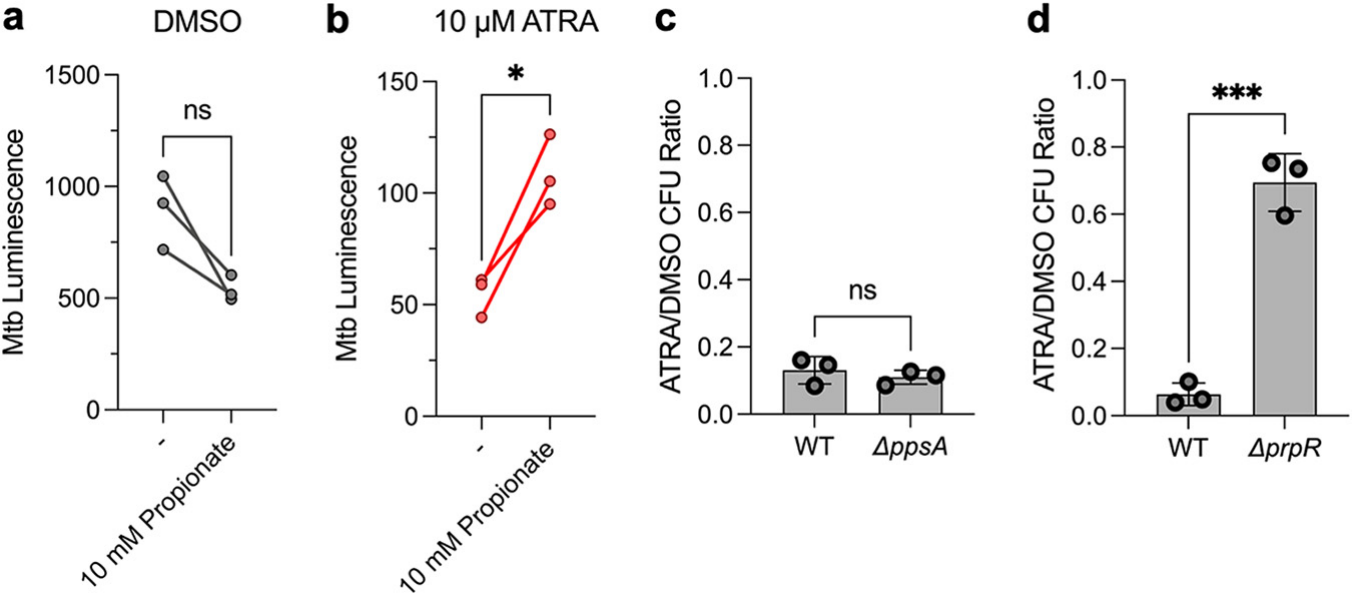
ATRA-mediated restriction is dependent on limited propionyl-CoA and the methylcitrate cycle. (a, b) MO-GMCSF from 3 donors were infected with autobioluminescent Mtb at a multiplicity of infection of 2 bacteria per macrophage and treated with ATRA at 10 μM and/or propionate at 10 mM, with a baseline of 0.1% DMSO; luminescence was measured at 5.5 days. (c, d) BMDM (*n* = 3) were infected with the indicated Mtb strains (H37Rv background) at a multiplicity of infection of 2 bacteria per macrophage, with Mtb load enumerated by determining numbers of CFU at 5.5 days. Data are displayed as a ratio of the number of ATRA-treated CFU divided by the number of DMSO-treated CFU. Statistics were performed using a paired (a, b) or unpaired (c, d) *t* test. *, *P* < 0.05; ***, *P* < 0.001. WT, wild type.

The fact that supplementation with oleic acid, a commonly used carbon source for Mtb, does not abrogate restriction by ATRA suggests that FA starvation *per se* is not a primary cause of ATRA-induced macrophage control of Mtb. Notably, the catabolism of OCFAs but not ECFAs produces propionyl-CoA. Cholesterol is the primary source of propionyl-CoA for Mtb in macrophages; OCFAs, though detectable, are present at very low levels (34, 49). Taken together, these data suggest that ATRA treatment of macrophages restricts Mtb survival through propionyl-CoA starvation. Consistent with this model, propionate supplementation alone was also able to alleviate ATRA-mediated bacterial control, albeit not to the same extent as cholesterol or OCFAs (Fig. 5a and b). This difference is likely due to the relative inaccessibility of exogenous propionate to intramacrophage Mtb; bovine serum albumin (BSA)-conjugated FAs and cholesterol efficiently cross the cell membrane and accumulate in lipid droplets (which are then directly consumed by intracellular Mtb) (49–51), while propionate is transported inefficiently by passive diffusion (52).

### Propionyl-CoA starvation stresses anaplerosis and gluconeogenesis.

**FIG 6:**
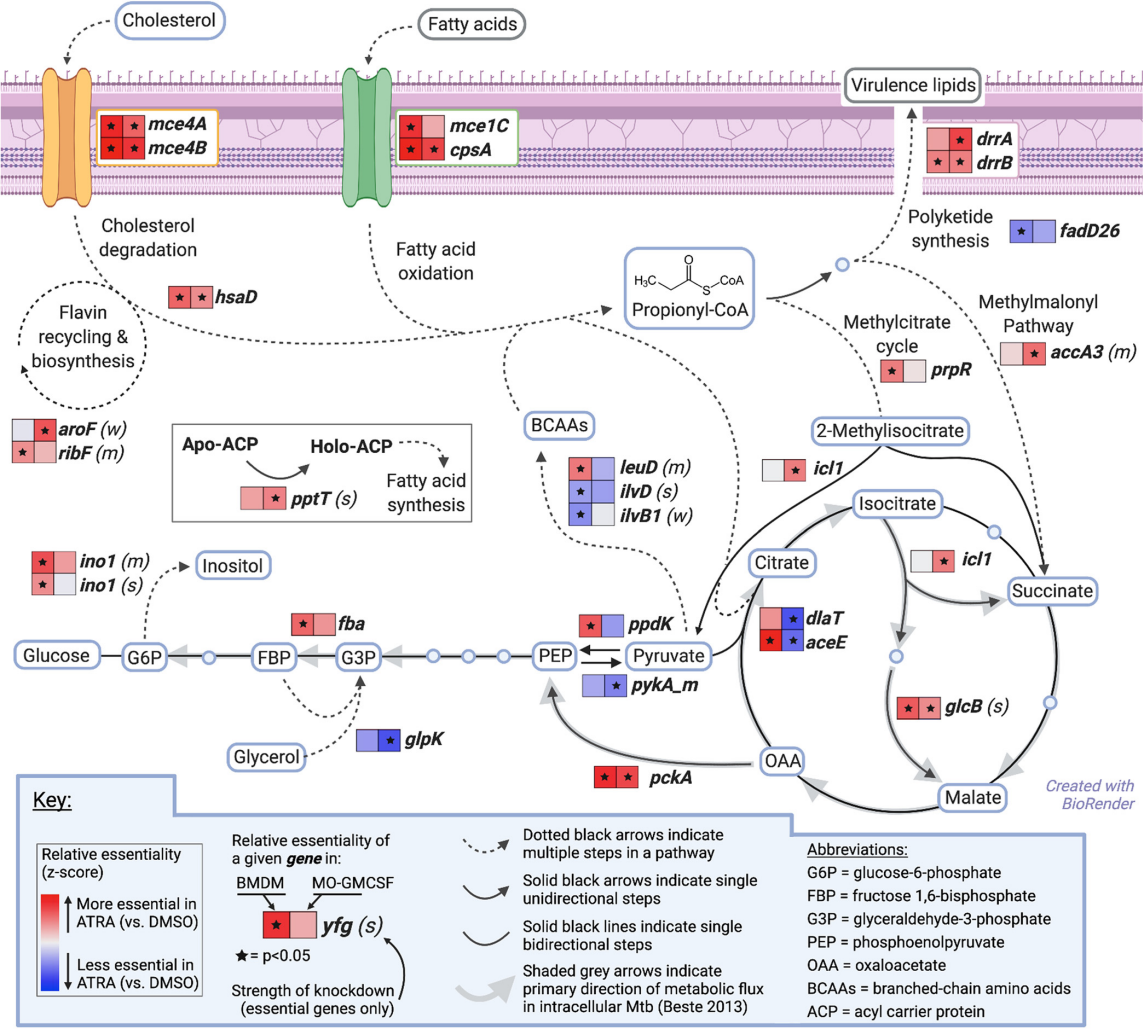
M. tuberculosis increasingly requires anaplerotic assimilation of propionyl-CoA within macrophages treated with all-*trans*-retinoic acid. Model of cholesterol and fatty acids crossing the Mtb cell wall (purple) and progressing through propionyl-CoA metabolism, with conditional essentiality during ATRA treatment of macrophages for selected Mtb genes shown in heatmaps (data are from Table S3). Specific molecules are in blue boxes, and molecular classes are in gray boxes. Metabolic flux directionality was adapted from the published literature (53). Statistics were performed with MAGeCK MLE analysis for Wald *P* values.

In Mtb, propionyl-CoA enters three major metabolic pathways (Fig. 6): generation of polyketide virulence-associated lipids such as phthiocerol-dimycocerosate (PDIM), integration into central carbon metabolism via the methylcitrate cycle (MCC), or entry into the methylmalonyl pathway (MMP) (34). To understand whether Mtb’s need for propionyl-CoA in ATRA-treated macrophages reflected an increased requirement for one of these downstream pathways, we mapped changes in genetic requirements identified through our CRISPRi screens onto these pathways, with directionality informed by published metabolic flux analysis of intracellular Mtb (53). Despite the critical role of PDIM in virulence, the requirement for the PDIM biosynthetic gene *fadD26* significantly decreased with ATRA treatment, although the PDIM transport genes *drrA* and *drrB* became more required, perhaps reflecting the toxic effects of disrupting a transporter rather than a requirement for the product itself. Given these discrepant phenotypes, we confirmed that ATRA treatment does not increase the requirement for the PDIM biosynthetic gene *ppsA* using a deletion mutant and enumerating bacterial control by determining numbers of CFU (Fig. 5c; Fig. S5a and b).

10.1128/mbio.03683-21.6 FIG S5 ATRA-mediated restriction is dependent on the methylcitrate cycle in BMDM. BMDM (*n* = 3) were infected with the indicated Mtb strains (H37Rv background) at a multiplicity of infection of 2 bacteria per macrophage, with Mtb load enumerated by determining the number of CFU at 5.5 days. (a) Experimental control for panel b; (c) experimental control for panel d. These plots represent the raw CFU data used to calculate the ratios in Fig. 5c and d. Download FIG S5, PDF file, 0.10 MB. Copyright © 2022 Babunovic et al. 2022 Babunovic et al. https://creativecommons.org/licenses/by/4.0/ This content is distributed under the terms of the Creative Commons Attribution 4.0 International license.

In contrast, we find that ATRA increases requirements for genes encoding components of the MCC in BMDM and the MMP in MO-GMCSF. We confirmed the key role for the MCC in BMDM using a strain with a deletion of its regulator, *prpR*, enumerating bacterial survival (Fig. 5d; Fig. S5c and d). Central carbon flux for intracellular Mtb favors anaplerosis and gluconeogenesis, and anaplerosis is essential for survival within macrophages (53, 54); this suggests a gluconeogenic fate for propionyl-CoA assimilated into the tricarboxylic acid (TCA) cycle by the MCC or the MMP. Consistent with this, we see an increased requirement for *icl1*, *glcB*, *pckA*, and *fba* in the setting of ATRA treatment and a decreased requirement for the glycolytic gene *pykA* (Fig. 6). Overall, these data show that cholesterol and downstream propionyl-CoA limitation by ATRA-treated macrophages limits bacterial growth and survival by increasing the stress on key anaplerotic and gluconeogenic processes.

## DISCUSSION

Numerous studies have proposed interventions and mechanisms by which macrophage Mtb restriction might be achieved (1, 2, 4–15). Systematic comparison of 26 macrophage activators identified ATRA as eliciting the most effective control, outperforming clinically promising host-directed therapies. ATRA also surpassed traditional activators such as gamma interferon (IFN-γ), in agreement with the challenges that other groups have faced in using IFN-γ to consistently stimulate the control of Mtb in human macrophages (1, 8, 9). The robust restrictive effect of ATRA in both classically and alternatively activated primary human macrophages from independent donors led us to further investigate its antibacterial mechanism.

Dissection of ATRA-mediated bacterial control implicated several macrophage pathways, many of which have previously been associated with Mtb infection and indeed may represent different facets of a coordinated axis. However, we have limited understanding of macrophages’ executioner functions that mediate Mtb clearance. In order to survive human immune assaults, mycobacteria necessarily must resist exposure to oxidative and nitrosative stresses, degradative enzymes, acidic pH, and starvation. While many studies, including those investigating ATRA and cholesterol limitation, associate bacterial control with increased phagolysosomal fusion or autophagy (5, 6, 29–32), Mtb extensively manipulates the macrophage environment (32, 55, 56), and indeed pathogenic mycobacteria can remain viable as they traffic into lysosomal vacuoles and then back to early endosomes (57).

We therefore generated a systematic understanding of bacterial restriction by leveraging the Mtb CRISPRi system in an infection model. This approach relies on the detection of quantitative changes in requirements for a given gene product, indicating which pressures created by ATRA are the most relevant to bacterial survival and its inhibition. Because it is possible to tune the strength of knockdown, CRISPRi provides a much greater quantitative range than mutant screens (42). However, it is worth noting some limitations. First, while we have developed rules to tune knockdown strength, we have not individually measured inhibition for every guide and thus can best interpret data from strains where knockdown changes fitness under at least one condition. Moreover, for essential genes, we can detect a quantitative change between macrophage conditions only when we achieve a Goldilocks level of knockdown, great enough to functionally reduce protein levels but not so large as to kill the organism regardless of macrophage state. Finally, the relationship between the expression of gene products and bacterial fitness may be nonlinear. Therefore, the magnitude of a fitness defect must be interpreted with some caution.

Recognizing these limitations, our data are striking in that several knockdowns indicate that ATRA treatment increases Mtb’s need for cholesterol and propionyl-CoA. Until now, the downstream role of ATRA-mediated cholesterol limitation in Mtb-infected macrophages was unknown; these data show that macrophage cholesterol limitation acts as nutritional immunity, directly depriving Mtb of a favored carbon source. The effects of this limitation—propagated through anaplerosis and gluconeogenesis rather than virulence lipid biosynthesis—impact not only bona fide gluconeogenic genes but also genes such as *ino1* that generate biomass from gluconeogenic substrates, as well as generalist biosynthetic genes like *pptT*. Interestingly, despite the apparent chokehold on gluconeogenic substrates, Mtb does not appear able to adapt by using glycerol as an alternative source, as we find a reduced requirement for *glpK* following ATRA treatment. It also does not display a clear increase in demand for branched-chain amino acid biosynthesis, though these compounds can serve as propionyl-CoA sources (34); this is consistent with starvation for gluconeogenic intermediates.

Further highlighting the importance of gluconeogenic flux, enzymes from the postglycolysis pyruvate dehydrogenase complex encoded by the genes *dlaT* and *aceE* are significantly less required for human macrophage survival during ATRA treatment. The *aceE* gene is, however, more required during ATRA treatment in murine BMDM; this is potentially due to its additional function in nitrosative-stress resistance (44), which is specifically important in murine macrophages (12). These data suggest involvement of secondary mechanisms of Mtb killing downstream of ATRA-mediated cholesterol and propionyl-CoA limitation. Importantly, secondary killing mechanisms may explain the autophagy dependence of ATRA-mediated restriction seen with the H37Ra strain of Mtb (6).

Limiting host macrophage cholesterol is a recognized therapeutic target for tuberculosis. Previous efforts have identified a cholesterol biosynthesis inhibitor, BM 15766, as effective against intramacrophage Mtb; moreover, there are widely used drugs targeting eukaryotic cholesterol metabolism, including statins, being assessed as host-directed therapies (30, 31). Interestingly, these two classes of cholesterol biosynthesis inhibitors differ in specifics of Mtb control: BM 15766, similarly to ATRA, is ineffective against a strain lacking *prpR* (which is required for Mtb to grow on cholesterol), while simvastatin restricts growth of the *prpR* mutant, consistent with the model that statins control Mtb via autophagy (30). Collectively, these data suggest that cholesterol limitation is a powerful lever for restricting the intracellular growth and survival of Mtb, with possible antimicrobial effects through macrophage effector mechanisms and through targeting the Mtb-macrophage-coupled metabolic system with bacterial propionyl-CoA starvation.

ATRA is used as a chemotherapeutic drug (24), and its ability to elicit Mtb control by macrophages supports RAR targeting as a therapeutic avenue for tuberculosis disease. There are, of course, challenges in extrapolating from success in cellular models to treatment of a whole animal or person. We designed our study using macrophages from different maturation conditions in an effort to model some features of the varied environments that Mtb encounters *in vivo*. In the lung, Mtb faces ontologically distinct alveolar macrophage populations, matured in a GMCSF-replete environment, as well as recruited monocyte-derived macrophages (28, 58, 59). *In vitro* models using blood- and bone marrow-derived cells are likely to best mimic the biology of the recruited populations, and it is notable that we saw little difference in the levels of effectiveness of the panel of activators between classically and alternatively activated macrophages. However, future animal studies will be required to assess the generalizability of these data to tissue-resident cells in the pulmonary microenvironment.

ATRA’s therapeutic use may be limited by its short half-life *in vivo* (60, 61), which may explain its variable magnitude of Mtb restriction in animal models (62–64). More stable drug delivery systems exist for ATRA (64, 65), as well as other possibilities for RAR targeting (66). However, as retinoic acid mimetics are explored therapeutically, it is worth noting the differences in effectiveness between ATRA, TTNPB, and EC23 in our studies. These differences may reflect quantitative differences in receptor engagement and response dynamics. However, qualitative differences in specific compound-receptor interactions have been described. For example, in some models, ATRA has been shown to activate not only RARs but also the nuclear receptor dimer PPARβ/δ, while TTNPB does not share this ability (67); the resulting differences in transcriptional responses may contribute to differences in therapeutic effect.

Finally, ATRA is also naturally and continually present in human cells as an active metabolite of vitamin A, which is important for resistance to a variety of bacterial, viral, and parasitic infections (68, 69). This is especially relevant to tuberculosis disease, as vitamin A deficiency has been strongly associated with tuberculosis progression in humans (70). Thus, this work not only supports ATRA as a therapeutic option but also deepens our understanding of how natural human immunity to tuberculosis can subvert Mtb metabolism.

## MATERIALS AND METHODS

For expanded Materials and Methods, see Text S1 in the supplemental material.

10.1128/mbio.03683-21.1 TEXT S1 Expanded materials and methods, with more granular experimental detail. Citations refer to the primary reference section of the paper. Download Text S1, PDF file, 0.07 MB. Copyright © 2022 Babunovic et al. 2022 Babunovic et al. https://creativecommons.org/licenses/by/4.0/ This content is distributed under the terms of the Creative Commons Attribution 4.0 International license.

### Reagents.

Cytokines were purchased from BioLegend. Purchased compounds were from Tocris Bioscience, MilliporeSigma, Cayman Chemical, InvivoGen, ThermoFisher, or Enzo Life Sciences. Fumonisin B1 was a gift from Fikadu Tafesse; the 19-kDa antigen was a gift from Robert Modlin. Complexes of fatty acids (FAs) and BSA were prepared at a 3:1 FA/BSA molar ratio in phosphate-buffered saline (PBS) and homogenized at 60°C for 20 min when necessary.

### Mammalian cell culture.

The medium for cell culture was RPMI 1640 with 10% fetal bovine serum (FBS), 10 mM HEPES, and 1× GlutaMAX (cRPMI). Primary human monocytes were isolated from peripheral blood mononuclear cells obtained by Ficoll gradient centrifugation of healthy donor leukaphereses (Research Blood Components) or buffy coat blood (Massachusetts General Hospital). Monocytes were isolated by CD14-positive selection (Stemcell Technologies) and matured in 50 ng/mL cytokine for 6 days. Matured human macrophages were dissociated with Accutase (Innovative Cell Technologies) and adhered overnight in medium without added cytokine.

Low-passage-number THP-1 monocytes (ATCC TIB-202) were adhered to treated plates with 50 ng/mL phorbol 12-myristate 13-acetate (PMA) (Calbiochem), followed by 24 h of rest. L929 fibroblasts (gift from Gökhan Hotamisligil) were passaged below confluence; to generate the L929 supernatant, cells were left at 100% confluence for 7 days. Murine bone marrow was isolated from 6- to 8-week-old female C57BL/6 mice (Jackson Laboratory). BMDM were matured in bacteriological petri dishes for 7 to 8 days in medium containing the L929 supernatant (25%). BMDM were dissociated at 4°C with 2 mM EDTA in PBS with scraping and adhered overnight. BMDM were maintained in medium containing the L929 supernatant (10%).

### Bacterial strains and growth conditions.

Mycobacteria were grown in Middlebrook 7H9 medium (BD) supplemented with 10% oleic acid albumin-dextrose-catalase (OADC) (BD), 0.2% glycerol, and 0.05% Tween 80 (complete 7H9), with antibiotics when appropriate. Mtb Δ*prpR* (71) and Δ*ppsA* were gifts from Christopher Sassetti. Mtb H37Rv-lux was-generated using a modified form of pMV306hsp+LuxG13 (Addgene 26161) (22). Culture density was measured by determining the optical density at 600 nm (OD_600nm_) and/or autobioluminescence on a BioTek Synergy H1 reader.

### Bacterial infection and analysis.

Mycobacteria grown in complete 7H9 were pelleted by centrifugation and prepared in cRPMI by either 5-μm filtration or soft spinning. Soft spinning is a modified published method (72). Bacteria were washed, pelleted, resuspended, and centrifuged for 8 min at 121 × *g*, with the top half of the centrifuged suspension used. Suspensions were diluted to a multiplicity of infection of 1 to 2 bacteria per macrophage by determining the OD_600nm_. Macrophages were infected for 4 to 16 h, followed by a PBS wash and application of the experiment medium.

For CFU, supernatants and adherent cells were separately lysed in 0.1% Triton X-100 in PBS. Samples were diluted and plated onto Middlebrook 7H11 agar with OADC (BD) for enumeration. Autobioluminescence was measured using a BioTek Synergy H1 reader. For human macrophages, measurements were normalized donor by donor to the final untreated time point.

Cholesterol levels were measured with the cholesterol Ester-Glo kit (Promega), prepared according to the manufacturer’s instructions, and measured using a BioTek Synergy H1 reader. Measurements were converted to micromolar units using GraphPad Prism and normalized donor by donor to the untreated condition.

For Seahorse analysis, MO-GMCSF in an XF24 plate infected with M. bovis BCG were switched to experiment medium for 44 h. This plate was analyzed with the Seahorse XF Cell Mito stress test kit (Agilent Technologies) on a Seahorse XFe24 analyzer according to the manufacturer’s instructions.

### Transcriptional analysis.

Cells were lysed 24 h (THP1) or 11 h (MO-GMCSF) after treatment in TRIzol (ThermoFisher) or Buffer RLT with β-mercaptoethanol (Qiagen), respectively. RNA was purified using the Zymo Direct-Zol kit according to the manufacturer’s instructions, with off-column DNase treatment and repurification.

For quantitative PCR (qPCR), cDNA was generated using SuperScript IV (ThermoFisher) and random hexamers and quantified with iTaq Universal SYBR green supermix (Bio-Rad) using 400 nM each primer (oligonucleotides 1 to 10 in Table S4 in the supplemental material) (73) on an Applied Biosystems ViiA 7 system. Gene expression was normalized to that of GAPDH (glyceraldehyde-3-phosphate dehydrogenase). For RNA-seq, libraries were prepared using the KAPA mRNA HyperPrep kit with KAPA dual indexed adapters (Roche) according to the manufacturer’s instructions. Each sample was sequenced to a depth of ∼1 × 10^7^ single-end reads using a NextSeq 75 cycle high-output kit (Illumina).

10.1128/mbio.03683-21.10 TABLE S4 Oligonucleotides used in this study. Individually ordered DNA oligonucleotides and pooled members of the CRISPRi oligonucleotide library are displayed in separate sheets. Download Table S4, XLSX file, 0.03 MB. Copyright © 2022 Babunovic et al. 2022 Babunovic et al. https://creativecommons.org/licenses/by/4.0/ This content is distributed under the terms of the Creative Commons Attribution 4.0 International license.

Reads were aligned using the STAR aligner within the RSEM program (74). Counts from 3 donors with high correlation were analyzed using DESeq2 in R (75). apeglm-shrunk (76) log_2_ fold changes were analyzed with a significance cutoff at an adjusted *P* value of <0.05 and an absolute fold change of >1.5. For network plotting, normal-shrunk log_2_ fold changes were used in preranked-list weighted gene set enrichment analysis (GSEA) against all gene sets from the hallmark, canonical pathway, and gene ontology (GO) collections in MSigDB (version 7.1), with at least 3 and fewer than 500 genes (77, 78). The EnrichmentMap plugin for Cytoscape (79) was used to plot gene sets enriched (false-discovery rate [FDR] < 0.1 and *P* < 0.05) in both comparisons with enough shared genes to form a gene set cluster.

### Preparation of bacterial CRISPRi library and clonal strains.

For essential genes (41), sgRNA sequences were selected representing a “weak,” “medium,” and/or “strong” knockdown, based on sgRNA dropout during induced culture (42). For nonessential genes, sgRNAs were selected based on proximity to high-knockdown protospacer adjacent motif (PAM) sites (40). Selection prioritized PAM site diversity and avoided transcriptional start sites (80). Negative guides were selected for stability of representation in induced culture.

Seven hundred twenty-six targeting and 50 overrepresented nontargeting guides (Table S2) were purchased as DNA oligonucleotides (GenScript) (Table S4). Pooled oligonucleotides were amplified (primers were oligonucleotides 11 and 12) and column purified (Qiagen). Plasmid plJR966 (40) was isolated using the Plasmid *Plus* maxikit (Qiagen). Oligonucleotides were ligated into BsmBI-digested plJR966 (after gel purification [Qiagen] and acetate-ethanol precipitation) via golden gate cloning. The ligation product was column purified (Zymo) and dialyzed.

The ligation product was electroporated into MegaX DH10B T1 cells (Thermo), with coverage of ∼1,500× per targeting sgRNA. Plasmid was isolated from resuspended colonies using the Plasmid *Plus* maxikit (Qiagen). The plasmid library was electroporated into Mtb H37Rv (grown in 200 mM glycine, followed by a glycerol wash), with coverage of ∼750× per targeting sgRNA and with recovery in complete 7H9 followed by selection for 20 days on complete 7H10 agar with kanamycin. Liquid Mtb culture was partially outgrown from colonies in complete 7H9.

Luminescent clonal CRISPRi strains were generated by golden gate ligation of individual amplified oligonucleotides (oligonucleotides 13 to 19 in Table S4) into BsmBI-cut plJR966. Plasmids were electroporated into Mtb H37Rv-lux and selected on complete 7H10 agar with kanamycin and zeocin. Plasmids for negative guides were isolated from colonies of the pooled CRISPRi library.

### Bacterial CRISPRi screening.

CRISPRi libraries were grown with kanamycin and used to infect macrophages. Soft-spun bacteria (inoculum) were also used to start axenic cultures in complete 7H9 media with kanamycin, with or without an anhydrotetracycline (ATc) inducer, and grown for 8 days with a single dilution. BMDM and MO-GMCSF were infected in separate experiments. Following 7 days of infection in media containing ATc and DMSO or ATRA, macrophages were lysed and equally outgrown in complete 7H9 with kanamycin.

Following lysis by bead beating, genomic DNA was isolated using phenol-chloroform extraction. Sequencing libraries were amplified by PCR with dually indexed primers (oligonucleotides 20 to 39 in Table S4) and gel purified (Qiagen). Libraries were single-end sequenced using NextSeq 150-cycle mid-output kits (Illumina).

Reads were aligned using a Python script filtering for perfect PAM and sgRNA matches, and counts were analyzed using the MAGeCK maximum likelihood estimation (MLE) program (81). Essential gene hypomorphs of different strengths were analyzed as separate “genes.” sgRNAs for each nonessential gene were analyzed together. Negative sgRNAs were either considered individual “genes” (when normalizing to total counts) or a single “gene” (when normalizing to negative-control sgRNAs).

### Approval.

Human primary cells were obtained with the approval of the Harvard Longwood Campus Institutional Review Board. Mice were handled according to protocols approved by the Harvard Medical Area Standing Committee on Animals.

### Data availability.

All RNA sequencing data are available via the NCBI Gene Expression Omnibus (https://www.ncbi.nlm.nih.gov/geo) under accession number GSE183912. Software used in this study, as detailed and cited above, include STAR aligner (version 2.6.0), RSEM (version 1.2.29), R (version 3.6.3; https://www.r-project.org/), DESeq2 (version 1.24.0), Python (version 2.7.17; https://www.python.org/), MAGeCK (version 0.5.9.4), and Cytoscape (version 3.8.2; https://cytoscape.org/).
