# Supplementary figures and images for: CRISPR Interference Reveals That All-Trans-Retinoic Acid Promotes Macrophage Control of Mycobacterium tuberculosis by Limiting Bacterial Access to Cholesterol and Propionyl Coenzyme A
Source: mBio. 2022 Jan 18;13(1):e03683-21. doi: 10.1128/mbio.03683-21 (PMC8764544; doi:10.1128/mbio.03683-21)

**a**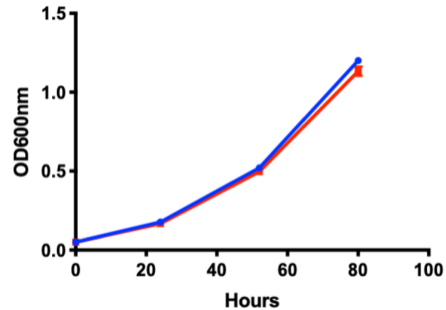**b**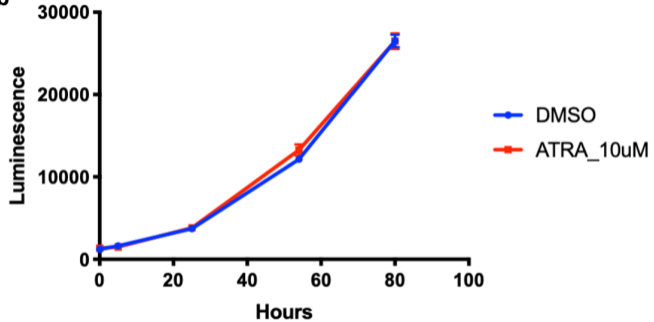

Supplement: FIG S1 [file mbio.03683-21-sf001.pdf]

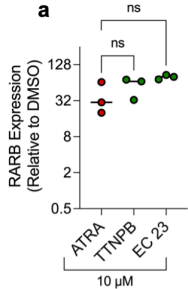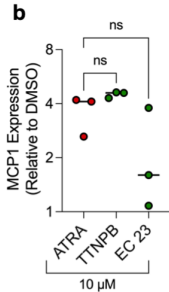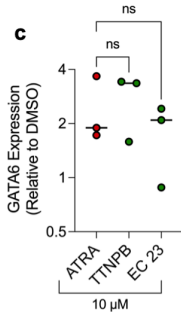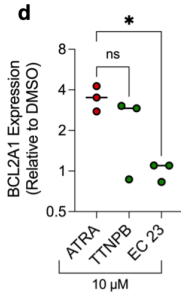

Supplement: FIG S2 [file mbio.03683-21-sf002.pdf]

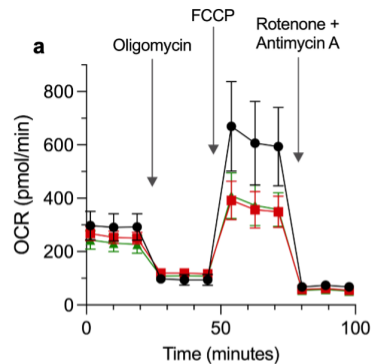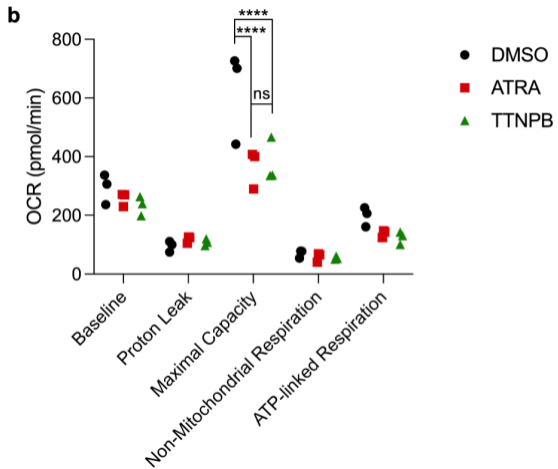

Supplement: FIG S3 [file mbio.03683-21-sf003.pdf]

Mtb Luminescence

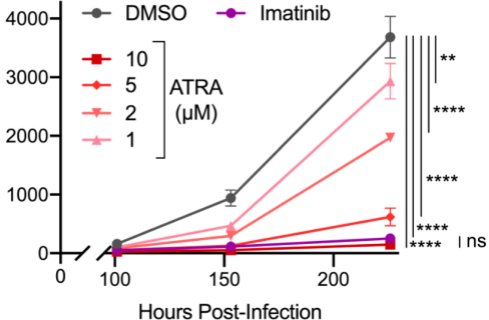

Supplement: FIG S4 [file mbio.03683-21-sf004.pdf]

**a**

WT

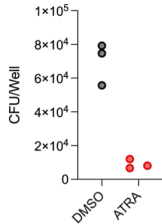**b** $\Delta ppsA$ 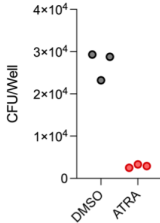**c**

WT

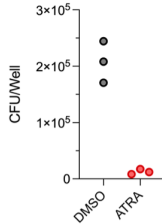**d** $\Delta prpR$ 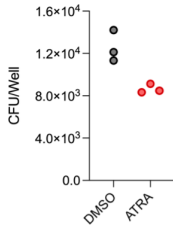

Supplement: FIG S5 [file mbio.03683-21-sf005.pdf]
