# Supplementary material for: CRISPR Interference Reveals That All-Trans-Retinoic Acid Promotes Macrophage Control of Mycobacterium tuberculosis by Limiting Bacterial Access to Cholesterol and Propionyl Coenzyme A
Source: mBio. 2022 Jan 18;13(1):e03683-21. doi: 10.1128/mbio.03683-21 (PMC8764544; doi:10.1128/mbio.03683-21)
Supplement: TEXT S1 [file mbio.03683-21-s0001.pdf]

Appendix – Expanded Materials and Methods

This appendix contains a comprehensive and more detailed description of all materials and methods used in this study.

Reagents

All-*trans*-retinoic acid was purchased from Tocris Bioscience. Water-soluble cholesterol, ATP, prostaglandin E2, retinol, oleic acid, pentadecanoic acid, essentially fatty acid-free bovine serum albumin (BSA), imatinib mesylate, gefitinib, and anhydrotetracycline were purchased from MilliporeSigma. TTNPB and EC23 were purchased from Cayman Chemical. Human M-CSF, GM-CSF, IFNγ, TNFα, TGFβ, IL-4, IL-6, IL-8, IL-10, and IL-13 were purchased from BioLegend. Pam3CSK4 was purchased from InvivoGen. Oxidized LDL was purchased from ThermoFisher. 1α,25-dihydroxyvitamin D3 was purchased from Enzo Life Sciences. Fumonisin B1 was a gift from Fikadu Tafesse, and 19 kDa antigen was a gift from Robert Modlin.

All small molecules were dissolved in DMSO (MilliporeSigma) or purified distilled water, and sterilized by 0.2 μm filtration before use. Complexes of fatty acids (FAs) and BSA were prepared by first adding 10 mM FA to 3.3 mM BSA in phosphate-buffered saline (PBS). For pentadecanoic acid, and for oleic acid when being directly compared to pentadecanoic acid, FA-BSA complexes were homogenized/clarified by heating at 60°C for 20 minutes with mixing; otherwise, oleic acid complex was mixed at 37°C until homogeneous and clear. These complexes were filter-sterilized prior to use.

Mammalian cell culture

All cells were maintained in cRPMI: RPMI with 10% performance plus FBS (heat-inactivated for 30 minutes at 56°C), 10 mM HEPES, and 1x GlutaMAX (all purchased from ThermoFisher). All incubations were performed at 37°C with 5% CO_2. All macrophages were plated at a final density of 1-1.3x10^5 cells/cm^2 for experiments.

Primary human monocytes were isolated from frozen peripheral blood mononuclear cells obtained by Ficoll gradient centrifugation of of healthy donor leukaphereses (Research Blood Components) or buffy coat blood (Massachusetts General Hospital) following approval of the protocol by the Harvard Longwood Campus Institutional Review Board. Monocytes were isolated by CD14 positive selection (Stemcell Technologies), and matured into macrophages on tissue culture treated dishes using 50 ng/mL GM-CSF or M-CSF for 6 days. Matured human macrophages (MO-MCSF or MO-GMCSF) were dissociated with accutase (Innovative Cell Technologies) followed by pipetting and scraping, counted, distributed in tissue culture treated plates, and allowed to adhere overnight in the same media without added cytokine.

THP-1 monocytes (ATCC TIB-202) were passaged from ~2x10^5 to ~1x10^6 cells/mL; prior to experiments, they were adhered to tissue culture treated plates by 24h of treatment with 50 ng/mL phorbol 12-myristate 13-acetate (PMA) (Calbiochem) followed by 24h of incubation without PMA. L929 murine fibroblasts (gift from Gökhan Hotamisligil) were passaged from ~1x10^4 cells/cm^2 to ~80% confluence, with 0.25% trypsin-EDTA (Gibco) used to detach cells at each passage; to generate L929 conditioned supernatant, cells were left at 100% confluence for 7 days before the culture supernatant was decanted, filtered, and stored at -80°C.

Murine bone marrow was isolated from the femurs and tibias of 6-8 week old female C57BL/6 mice (Jackson Labs) by centrifugation of bones with the ends cut open (all animal work was approved by the Harvard Medical Area Standing Committee on Animals). Bone marrow cells were matured to BMDM in bacteriological petri dishes at ~7x10^4 cells/cm^2 (excluding red blood cells) for 7-8 days, with media containing 1x anti-anti (Gibco) and 25% L929 supernatant. Following maturation, BMDM were dissociated by treatment at 4°C with 2 mM EDTA in PBS followed by scraping, counted, distributed in tissue culture treated plates or dishes, and allowed to adhere overnight. BMDM were continually maintained in media containing 10% L929 supernatant.

Bacterial Strains and Growth Conditions

All strains of Mtb H37Rv and *Mycobacterium bovis* BCG were grown in Middlebrook 7H9 medium (BD) supplemented with 10% oleic acid albumin-dextrose-catalase (OADC) (BD), 0.2% glycerol, and 0.05% Tween-80 at 37°C (complete 7H9). When appropriate, 20 µg/mL kanamycin and/or or 20 µg/mL zeocin were added to the growth medium. Mtb Δ*prpR*, used previously (71), and Mtb Δ*ppsA* were gifts from Christopher Sassetti. Mtb H37Rv-lux was generated by transformation of a modified form of pMV306hsp+LuxG13 (Addgene 26161) (22), with the kanamycin resistance cassette replaced with a zeocin resistance gene and the L5 integration machinery replaced with the same components targeting the Tweety site. Bacterial density in axenic culture was measured by OD_{600nm} and/or by autobioluminescence of samples in a white-bottom 96-well plate (Corning) read by a BioTek Synergy H1 microplate reader.

Bacterial infection of mammalian cells

Strains of Mtb or *M. bovis* BCG were grown in complete 7H9 medium as described above to mid-log phase (OD_{600nm} of ~0.4), then pelleted by centrifugation and prepared for infection by either filtration or soft spin to remove bacterial clumps (used interchangeably with equivalent results). For filtration, pelleted bacteria were resuspended in the appropriate mammalian cell culture media and filtered gently through a 5 µm filter. For soft spin we implemented a modified version of a published method (72), wherein pelleted bacteria were washed once with an equal volume of PBS, pelleted again, resuspended in the appropriate mammalian cell culture media, and then centrifuged for 8 minutes at 121xg; the top half of the undisturbed post-centrifugation suspension (3-5 mL of 6-10 mL) was removed and used for infection.

Following either filtration or soft spin, clump-free suspensions were measured for OD_{600nm} and diluted to the appropriate final titer for a multiplicity of 1-2 bacteria per macrophage (an OD_{600nm} value of 1.0 corresponds with a titer of $3x10^8$ cfu/mL). Media was aspirated from macrophages and replaced with these dilute bacteria for 4-16 hours, after which the macrophages were washed 3 times with PBS and the final experimental media (containing any compounds or activators) was applied.

CFU enumeration, cholesterol measurement, and luminescence

Prior to lysing macrophages for Mtb CFU enumeration, infected cells at the stated timepoint were separated from supernatants and gently washed once with PBS. This PBS was then removed, mixed with the supernatant, and combined with Triton X-100 to a final concentration of 0.1%, to create a "dissociated" fraction. Meanwhile, adherent macrophages after washing were lysed with 0.1% Triton X-100 in PBS for at least 5 minutes, followed by vigorous pipetting to homogenize lysates. Lysates and the dissociated fraction were further diluted in PBS with 0.05% tween-80, plated onto Middlebrook 7H11 agar with OADC (BD), and incubated at 37°C for 11-16 days prior to enumeration at least twice (at least 2 days apart) for each sample. Final CFU counts represent the total number of bacteria (combined lysate and dissociated fraction) in one well of a 96-well plate.

For determining Mtb burden by autobioluminescence, macrophages were maintained and infected in white-bottom plates and luminescence was measured using a BioTek Synergy H1 microplate reader. For human macrophages, measurements were normalized on a donor-by-donor basis to the final timepoint of the untreated condition; this allowed statistical comparisons across multiple donors. For murine BMDM, raw luminescence values were used for all comparisons.

For cholesterol measurement, macrophages at 1 day of incubation following infection and compound treatment were lysed and cholesterol was measured with the Cholesterol Ester-Glo™ kit (Promega) following the manufacturer's instructions. Briefly, macrophages were washed 2 times with PBS and lysed in the provided solution for 30 min. at 37°C, then diluted into prepared detection reagent (with esterase) and incubated for 1 hour at room temperature. Cholesterol standard was initially diluted in glass, then diluted into the final plate in a 2-fold series. Luminescence was measured using a BioTek Synergy H1 microplate reader; measurements were converted to µM using a standard curve interpolated in GraphPad Prism, and normalized to the untreated condition on a donor-by-donor basis.

Seahorse analysis

Macrophages (MO-GMCSF) in a Seahorse XF24 plate infected with *M. bovis* BCG for 5 hours were switched (without a PBS wash) to media containing DMSO, 10 µM ATRA, or 10 µM TTNPB for 44 hours. This plate was then analyzed for oxygen consumption rate with the Seahorse XF Cell Mito Stress Test Kit (Agilent Technologies) according to the manufacturer's instructions. Briefly, infected cells were washed with warm Seahorse base media (DMEM), then incubated for one hour in the same media with drugs in a humidified environment with ambient CO_2. Pre-warmed Seahorse media containing solubilized kit compounds—oligomycin, carbonyl cyanide-4 (trifluoromethoxy) phenylhydrazone (FCCP), and rotenone with antimycin A—was added to an overnight-hydrated cartridge. This cartridge was used for calibration and OCR measurement on a Seahorse XFe24 instrument.

RNA isolation, qPCR, and sequencing

For adherent THP1 monocytes, cells were lysed at 24 hours after compound treatment with TRIzol (ThermoFisher). For infected MO-GMCSF, cells were lysed at 11 hours after compound treatment with Buffer RLT (Qiagen) with 1% β-mercaptoethanol; lysates were then pipetted thoroughly to homogenize and double-filtered through 0.2 µm membranes to sterilize. RNA was purified for all lysates using the Zymo Direct-Zol kit according to the manufacturer's instructions. Briefly, lysates (in either TRIzol or RLT) were mixed with 1 volume 100% ethanol and bound to Zymo-Spin columns; columns were washed with Direct-zol™ RNA PreWash and RNA wash buffer followed by elution in water. Purified RNA was then treated with TURBO DNase (Invitrogen), and re-purified using the Zymo RNA Clean & Concentrator 5 kit according to the manufacturer's instructions.

For qPCR, cDNA was generated from purified THP1 RNA using SuperScript IV reverse transcriptase (ThermoFisher) with priming by random hexamers. Diluted cDNA was quantified for genes of interest using iTaq Universal SYBR Green Supermix (Bio-Rad) with 400 nM each primer (oligos #1-10, **Table S4**) (73) on an Applied Biosystems ViiA 7 thermocycler. All experimental gene expression values (calculated from Ct) were normalized to GAPDH expression before comparison between samples.

For RNA-seq, libraries were prepared from purified MO-GMCSF RNA using the KAPA mRNA HyperPrep kit with KAPA Dual-Indexed Adapters (Roche) according to the manufacturer's instructions. Briefly, purified RNA (40-150ng per sample) of high quality (RIN > 8) was used as input for mRNA capture on beads. Purified mRNA was fragmented by heating in the presence of magnesium for 7 minutes at 94°C, then reverse transcribed and A-tailed prior to adapter ligation with a 1.5 µM adapter stock concentration; after post-ligation cleanup, ligated cDNA was amplified for 13 cycles and cleaned up again to yield final libraries. Library DNA concentration was measured by Qubit dsDNA BR assay (ThermoFisher) and the KAPA Library Quantification Kit (Roche); fragment size was measured by HS D1000 TapeStation (Agilent). Libraries were sequenced to a depth of ~$1x10^7$ reads per sample using a NextSeq 75 cycle High Output kit (Illumina) in a single-end format.

RNA-seq reads were aligned to genome build hg38 using the STAR aligner within the RSEM program (74). Counts from 4 donor samples were analyzed using DESeq2 in R (75), and samples from 3 donors (18k, 18o, 19a) with high correlation were used in further analyses. For analysis of individual gene expression changes, log2 fold-changes were shrunk using the apeglm algorithm(76), with a significance of cutoff of an adjusted p-value of <0.05 and an absolute fold-change of >1.5 (log2 fold-change >0.585). For network analysis, log2 fold-changes shrunk with the normal algorithm were matched to gene names as HGNC symbols and exported from DESeq2. These were used as input into the Broad Institute gene set enrichment analysis (GSEA) program as a preranked list for all measured genes (77, 78). Weighted GSEA was performed for the ATRA/TTNPB and ATRA/EC23 comparisons against a collection of all gene sets from the hallmark, canonical pathway (Kegg, BioCarta, PID, and Reactome), and gene ontology (GO) collections in mSigDB (version 7.1) with at least 3 and fewer than 500 genes. For network plotting, GSEA results were imported into the EnrichmentMap plugin for Cytoscape (79); gene sets were included in the final network plot if they were significantly enriched (positively or negatively, FDR<0.1 and p<0.05) in both comparisons and they shared enough genes with another set to form a cluster (at least one connecting edge on a medium-dense connectivity setting). Leading edge genes were those within a given gene set cluster—present in at least one of the defining gene sets—which had the largest absolute log2 fold-change values.

Preparation of bacterial CRISPRi library and clonal strains

Selected genes were classified as essential (i.e. essential or growth defect) or nonessential based on published transposon insertion sequencing data (41). For essential genes, 6-9 sgRNA sequences were selected, 3 each representing “weak”, “medium”, and (for most genes) “strong” strength of knockdown, based on magnitude of sgRNA dropout following approximately 17 generations of induced axenic culture (42). Bins were determined as follows: strong sgRNAs were those with the absolute lowest representation following induced culture, generally representing less than 5% of total sgRNAs with a log2 fold-change in representation below -1.8 following 17 generations in axenic culture. Weak sgRNAs were those with little or no selection at 17 generations, and medium sgRNAs were those with intermediate reduced representation. For nonessential genes, up to 6 sgRNAs were selected based on proximity to high-knockdown protospacer adjacent motif (PAM) sites (40). Guides for all genes were selected to prioritize diversity of PAM sites, and filtered to avoid close proximity to transcriptional start sites (80). Negative guides were selected based on stability of representation in induced axenic culture.

Once selected, 726 targeting and 50 non-targeting guides (**Table S2**) were purchased as synthesized DNA oligos flanked by BsmBI sites and PCR handles (GenScript) (**Table S4**); each non-targeting guide was 10-fold more represented than each targeting guide. 4 pmol of this oligo library was amplified for 11 cycles using Q5 polymerase (NEB) and oligos #11 & 12 (**Table S4**) and purified by column cleanup (Qiagen). Plasmid pIJR966 (pIRL2) (40) was isolated using the Plasmid *Plus* Maxi kit (Qiagen) and digested completely with BsmBI (NEB), followed by gel purification (Qiagen) and precipitation with 300 mM sodium acetate (pH 5.2) followed by addition of ethanol to 70% v/v and overnight incubation at -20°C. Plasmid was then pelleted and washed 2 times with 70% v/v ethanol before final aqueous resuspension. Library was ligated into BsmBI-digested pIJR966 via golden gate cloning, using 5% v/v FastDigest Esp3I (Thermo) with FastDigest buffer, 10 mM DTT, 10 mM ATP, 10 units/μL T4 DNA ligase (NEB), and amplified oligo library with digested plasmid at a ~7:1 molar ratio (46 nM total). Reaction was performed with 50 cycles of 5 minutes at 37°C and 5 minutes at 16°C, followed by one hour at 55°C, followed by addition of another 2.5% v/v FastDigest Esp3I and incubation at 37°C for one hour prior to deactivation at 80°C. Golden gate reaction product was purified on a clean & concentrator 5 column (Zymo) and dialyzed on a Millipore VSWP membrane (0.025 μm pore size).

500 ng purified golden gate product was transformed via electroporation (2000 V, 25 μF, 200 Ω) into MegaX DH10B $T1^R$ cells (Thermo) at an efficiency of ~4000 colonies/ng, for library coverage of ~1500x for each targeting sgRNA. Scraped colonies were mixed approx. 1:1 with lysogeny broth media until homogeneous; plasmid library was isolated from this mixture using the Plasmid *Plus* Maxi kit (Qiagen). 1 μg plasmid library was transformed via electroporation (2500 V, 25 μF, 1000 Ω) into Mtb H37Rv at an efficiency of ~1000 colonies/ng, for a library coverage of ~750x for each targeting sgRNA. Mtb were rendered electrocompetent by washing with 10% glycerol following 24 hours of growth in complete 7H9 containing 200 mM glycine. Electroporated Mtb were recovered in complete 7H9 for 24 hours followed by incubation for 20 days on 7H10 agar (with 10% OADC, 0.5% glycerol, and 20 μg/mL kanamycin). Colonies were incubated for 3 days in complete 7H9 to generate turbid liquid culture, which was separated from remaining solid Mtb, supplemented with glycerol to 0.5%, and stored at -80°C until use.

Luminescent clonal CRISPRi strains were generated with an abbreviated version of the protocol outlined above. Briefly, individual oligos (#13-19, **Table S4**) were amplified and cloned using the golden gate strategy into BsmBI-cut pIJR966; these reaction products were directly transformed into DH5α cells (NEB), and the resulting miniprepped plasmids (Qiagen) were transformed by electroporation into Mtb H37Rv-lux and recovered on 7H10 media (BD, containing 10% OADC, 0.5% glycerol, and 20 μg/mL each kanamycin and zeocin). Individual plasmids for negative guides were isolated from single colonies of the pooled CRISPRi library. Individual colonies were grown to turbidity in complete 7H9 with kanamycin and zeocin; sgRNA sequence was confirmed using the CRISPRi library sequencing protocol described below.

Bacterial CRISPRi screening

Mtb CRISPRi libraries were grown to mid-log phase and used to infect macrophages as described above. Soft-spun bacteria were also used to start axenic cultures in complete 7H9 media with kanamycin, with or without anhydrotetracycline (ATc) inducer; excess soft-spun bacteria were pelleted and used as inoculum samples. BMDM were seeded and infected in T25 flasks, while MO-GMCSF were seeded and infected in a 24-well plate in a separate experiment. Following 7 days of infection in media containing ATc and DMSO or ATRA, BMDM were washed once with PBS and lysed with 0.1% triton X-100 in PBS, after which bacteria were pelleted, and resuspended bacteria were grown in complete 7H9 with kanamycin and without ATc for equal generations until turbid. Also after 7 days of infection, MO-GMCSF were lysed and grown in complete 7H9 until turbid. Axenic cultures were grown for 4 days, diluted, and grown for another 4 days.

All samples (from inoculum, BMDM, MO-GMCSF, and axenic culture) following selection and/or outgrowth were used for genomic DNA isolation: pelleted bacteria were lysed by bead beating (3x30 seconds at 6500 rpm in MP Lysing Matrix B tubes on an MP Benchtop Homogenizer) in Tris-EDTA (TE) buffer with phenol-choloroform (pH=8), and gDNA was extracted by the phenol-chloroform method followed by precipitation in cold isopropanol with sodium acetate. Sequencing libraries were amplified from 700 ng per sample of purified gDNA with an 18-cycle PCR protocol using NEBnext Ultra II Q5 Master Mix and dual-indexed primers with a staggered sequence for read 1 (#20-39, **Table S4**), and purified by pooled gel purification (Qiagen). Libraries were sequenced using NextSeq 150 cycle Mid Output kits (Illumina) in a single-end format.

Sequencing reads were aligned to sgRNA sequences using a custom script in Python, which identified both the PAM sequence for Sth1 dCas9 and the provided sgRNA sequences; it recorded a count only in cases of perfect alignment (no mismatches). Counts were analyzed using the MAGeCK MLE program in Python (81), with normalization to either total counts or negative control sgRNAs, and either inoculum or DMSO-treated samples as a baseline as indicated. For essential genes, hypomorphs at different strengths (strong, medium, or weak) were analyzed as separate “genes”. For nonessential genes, all sgRNAs were analyzed as a single gene. For negative guides, all sgRNAs were either analyzed as individual “genes” (when normalizing to total counts) or as a single “gene” (when normalizing to these negative control sgRNAs).
